# Functional Characterization of *GbERF13* Reveals Its Role in ABA-Responsive Fiber Development and Molecular Marker Development in Sea Island Cotton

**DOI:** 10.3390/plants15132074

**Published:** 2026-07-03

**Authors:** Jin Chen, Jinxuan Chen, Qingqing Yan, Min Gao, Qin Chen, Tao Lv, Quanjia Chen, Kai Zheng

**Affiliations:** 1 Xinjiang Key Laboratory of Crop Biological Breeding, College of Agriculture, Xinjiang Agricultural University, Urumqi 830052, China; 2 Crop Research Institute of Xinjiang Uygur Autonomous Region Academy of Agricultural Sciences, National Central Asian Characteristic Crop Germplasm Resources Medium-Term Gene Bank (Urumqi), Urumqi 830091, China

**Keywords:** Sea Island cotton, *GbERF13*, gene cloning, fiber development, haplotype analysis

## Abstract

Sea Island cotton (*Gossypium barbadense* L.) is a premium raw material for high-end textiles due to its excellent fiber quality. The AP2/ERF transcription factor family plays critical roles in plant growth and hormone signaling. Here, 161 *GbERF* family members were identified in Sea Island cotton and classified into nine subgroups, with *GbERF13* belonging to Group V. Expression analysis revealed that *GbERF13* was specifically and highly expressed in fibers, with transcript abundance peaking at 15–30 days post-anthesis (DPA), coinciding with the transition from fiber elongation to secondary wall thickening. Exogenous abscisic acid (ABA) treatment significantly induced *GbERF13* expression and inhibited fiber elongation. Heterologous overexpression of *GbERF13* in Arabidopsis increased trichome and root hair numbers while suppressing primary root growth, confirming its role in cell elongation and development. A nonsynonymous SNP (A/C) at the 117th base pair of the *GbERF13* coding region (*GbERF13*-117SNP) was identified in 213 Sea Island cotton accessions. Association analysis showed the C allele was significantly and positively associated with fiber length, strength, and uniformity. An allele-specific PCR marker was further developed for molecular breeding. Collectively, *GbERF13* acts as a key ABA-responsive transcription factor regulating fiber development, and its functional SNP marker provides a valuable tool for improving Sea Island cotton fiber quality.

## 1. Introduction

Cotton fiber is developed from the polar elongation of embryonic epidermal cells. The four developmental stages of initiation, elongation, secondary wall thickening and maturation jointly determine the core traits such as fiber length, strength and fineness, making cotton an important natural textile raw material [1,2]. Island cotton fiber has excellent quality and is the main raw material of high-end textiles [3]. Analysis of the molecular regulatory network of cotton fiber development is the key to high-quality cotton breeding [4,5,6].

AP2/ERF is a large plant-specific transcription factor family with a conserved AP2 domain, participating in hormone signaling, cell development and stress responses [7,8,9,10]. In cotton, multiple *ERF* members regulate fiber growth by mediating ethylene and auxin signals [11,12,13]. Previous studies identified a typical ethylene-dependent *GhEIN3*-*GhERF*-*COBL4* module controlling fiber development [14]. However, in multiple plant species, including Arabidopsis (*Arabidopsis thaliana* (L.) Heynh.) [15], tomato (*Solanum lycopersicum* L.) [16], maize (*Zea mays* L.) [17], and watermelon (*Citrullus lanatus* (Thunb.) Matsum. et Nakai) [18], numerous *ERF* members have been shown to participate broadly in ABA signal transduction.

Abscisic acid (ABA) is a key hormone involved in cotton fiber development. It can affect fiber development by inhibiting fiber elongation [19], promoting cell wall remodeling [20], regulating sucrose metabolism [21], and modulating secondary wall synthesis [4]. Cotton fibers, Arabidopsis leaf trichomes and root hairs are specialized single epidermal cells with highly conserved developmental regulatory pathways. Using Arabidopsis as a heterologous model is a well-established approach to studying cotton fiber-related genes. Given the low transformation efficiency and long growth cycle of Sea Island cotton, we adopted this classical heterologous expression system to conduct preliminary functional characterization of *GbERF13*. Molecular marker-assisted breeding is an important strategy for overcoming bottlenecks in the improvement of Sea Island cotton fiber quality. Haplotype analysis and functional marker development can directly associate natural variation in candidate genes with phenotypes [22,23,24]. Gene-level functional haplotype (FH) markers have been successfully used to elucidate the genetic mechanisms of cotton domestication and improvement [25]. Therefore, haplotype analysis and marker development based on the functional characterization of *GbERF13* can support rapid screening of breeding materials.

In follow-up research plans, we will construct overexpression and CRISPR/Cas9 knockout vectors to realize endogenous genetic transformation of sea island cotton and further verify the in vivo function of *GbERF13* in cotton fiber development.

The *GbERF13* gene was previously screened by QTL fine mapping technology and has been confirmed to be closely related to cotton fiber development. Based on this, we systematically studied its biological function. The purpose of this study was to analyze the evolutionary process of the *GbERF* family to elucidate the regulatory role of *GbERF13* in the development of Sea Island cotton fiber dependent on ABA signal, and to develop functional SNP markers suitable for molecular breeding. We propose two hypotheses: (1) *GbERF13* is a core ABA-responsive transcription factor; (2) the A/C non-synonymous mutation in the coding region of *GbERF13* affects fiber properties, and the C allele is a dominant allele associated with excellent fiber quality.

## 2. Results and Analysis

### 2.1. Identification and Expression Characteristics of the GbERF Family in Sea Island Cotton

#### 2.1.1. Identification and Evolution of the *GbERF* Family in Sea Island Cotton

**Figure 1:**
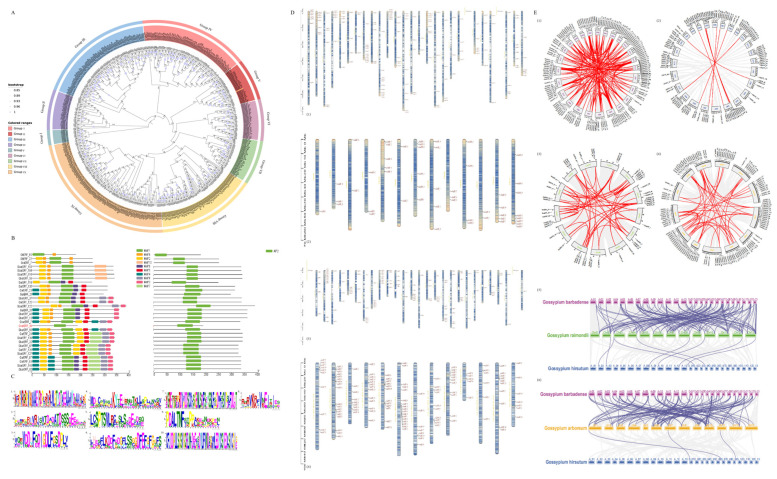
Phylogenetic tree and characteristic analysis of *ERF* family members. (**A**). Phylogenetic tree of *ERF* family members from four Gossypium species. Scale bar for phylogenetic tree = 0.1. (**B**). Visualization of conserved motifs and conserved domains in Group V subgroup members. (**C**). Motif signature sequences. (**D**). Chromosomal localization and distribution of the *ERF* gene family in different Gossypium species. Panels (1), (2), (3), and (4) show the chromosomal localization and distribution of *ERF* genes in Sea Island cotton, *G. raimondii*, upland cotton, and Asiatic cotton, respectively. (**E**). Intraspecific and interspecific collinearity analysis of the *ERF* gene family in four Gossypium species. Panels (1), (2), (3), (4), (5), and (6) show intraspecific collinearity in Sea Island cotton, intraspecific collinearity in upland cotton, intraspecific collinearity in *G. raimondii*, intraspecific collinearity in Asiatic cotton, interspecific collinearity among Sea Island cotton, *G. raimondii*, and upland cotton, and interspecific collinearity among Sea Island cotton, Asiatic cotton, and upland cotton, respectively.

In this study, 161 *GbERF* family members were identified in Sea Island cotton, whereas 49, 124, and 136 members were identified in G. raimondii, upland cotton, and Asiatic cotton, respectively. The phylogenetic tree divided all *ERF* family members into nine groups (Groups I–IX), among which *GbarERF89* (gene name: *GbERF13*) belonged to the Group V subgroup (Figure 1A). Conserved domain analysis showed that all members contained a complete AP2 domain (Figure 1B). Motif 1 and Motif 2 were the core conserved motifs and formed the framework of the AP2 domain (Figure 1C), whereas Motifs 3, 5, and 6 were key motifs associated with functional differentiation (Table 1). Chromosomal localization indicated that *GbERF* genes were unevenly distributed across the At/Dt subgenomes and were highly homologous to those of diploid ancestral cotton species (Figure 1D). Collinearity analysis confirmed that segmental duplication was the major driver of *GbERF* family expansion, with only limited gene loss and chromosomal rearrangement occurring during polyploidization (Figure 1E).

#### 2.1.2. Expression Characteristics of the *GbERF* Family in Sea Island Cotton

**Figure 2:**
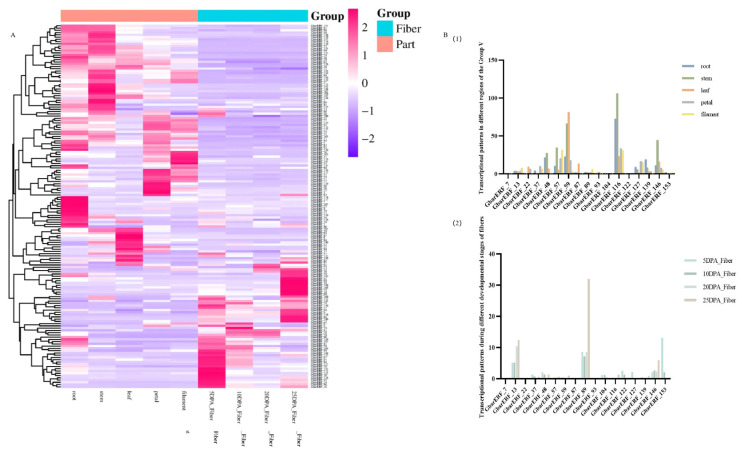
Transcriptional expression of the *GbERF* family in Sea Island cotton. (**A**). Transcriptional heatmap of *GbERF* gene family members in Sea Island cotton. (**B**). Transcriptional patterns of Group V subgroup members in different cotton tissues and at different fiber developmental stages. Panel (1) shows the transcriptional patterns of the subgroup members containing *GbERF13* in different cotton tissues; panel (2) shows the transcriptional patterns of the subgroup members containing *GbERF13* at different cotton fiber developmental stages.

The tissue expression profile showed that the *GbERF* family exhibited marked specificity, with multiple genes highly expressed in fibers at 10–25 DPA, suggesting their involvement in fiber elongation and secondary wall development (Figure 2A). Transcriptional analysis within the same subgroup showed that only a few genes were highly expressed under specific conditions. Genes highly expressed during fiber development (e.g., *GbarERF_13* and *GbarERF_89*) were generally expressed at low levels in various tissues, whereas genes highly expressed in tissues (e.g., *GbarERF_57*, *GbarERF_59*, and *GbarERF_116*) showed almost no transcription during fiber development, indicating clear functional differentiation (Figure 2B). *GbERF13*, namely *GbarERF_89*, was ultimately selected as the target for subsequent analysis.

### 2.2. Bioinformatics Analysis of the Sea Island Cotton GbERF13 Gene

**Figure 3:**
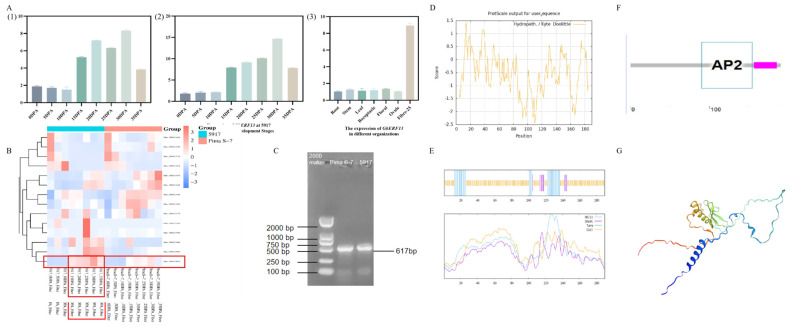
Cloning and bioinformatics analysis of the Sea Island cotton *GbERF13* gene. AP2/ERF denotes a large plant-specific transcription factor family defined by a conserved 60–70 amino acid AP2 DNA-binding domain [8]. (**A**). Expression levels of the Sea Island cotton *GbERF13* gene in different tissues and at different fiber developmental stages during cotton development. Panel (1) shows expression analysis of *GbERF13* in Pima S-7 cotton fibers at different developmental stages; panel (2) shows expression analysis of *GbERF13* in 5917 cotton fibers at different developmental stages; panel (3) shows expression analysis of *GbERF13* in different cotton tissues. (**B**). Heatmap of transcriptional expression of the Sea Island cotton *GbERF13* gene in different materials and at different fiber developmental stages during cotton development. (**C**). PCR amplification products of *GbERF13* cloning. (**D**). Hydrophilicity/hydrophobicity prediction. (**E**). Protein secondary structure. (**F**). Protein functional structure. (**G**). Protein tertiary structure prediction. All figures were exported at 300 DPI high resolution with enlarged axis labels and legends for clear online viewing.

In order to study the expression pattern of *GbERF13* gene in parental materials at different fiber development stages, real-time fluorescence quantitative PCR (qRT-PCR) analysis was carried out. The results showed that *GbERF13* had the highest expression level in cotton fiber tissues and extremely low expression in roots, stems, leaves and other tissues, showing fiber-specific high expression characteristics (Figure 3A). The results were also consistent with the transcriptome data to support this expression trend (Figure 3B). It indicated that *GbERF13* was expressed in both fiber elongation and secondary wall thickening stages. In this study, the *GbERF13* gene (Figure 3C) was cloned from *Gossypium barbadense*. The full-length coding sequence (CDS) of the *GbERF13* gene was 617 bp in length, encoding 188 amino acids. Bioinformatics analysis showed that the molecular weight of the encoded protein was 20,746.27 Da, and the isoelectric point was 9.15, which was an unstable hydrophilic protein (Figure 3D). Subcellular localization prediction showed that it was located in the nucleus (Table A1). The secondary structure analysis showed that the protein was mainly composed of random coil structure (77.37%), α-helix and extended strand accounted for 17.37% and 5.26%, respectively, and the β-turn accounted for 0% (Figure 3E). The protein also contains an AP2 domain (Figure 3F). These physicochemical properties and secondary and tertiary structural characteristics are consistent with the typical characteristics of ERF transcription factors (Figure 3G).

### 2.3. Transformation and Phenotypic Identification of the Sea Island Cotton GbERF13 Gene

#### 2.3.1. Transformation of the Sea Island Cotton *GbERF13* Gene

**Figure 4:**
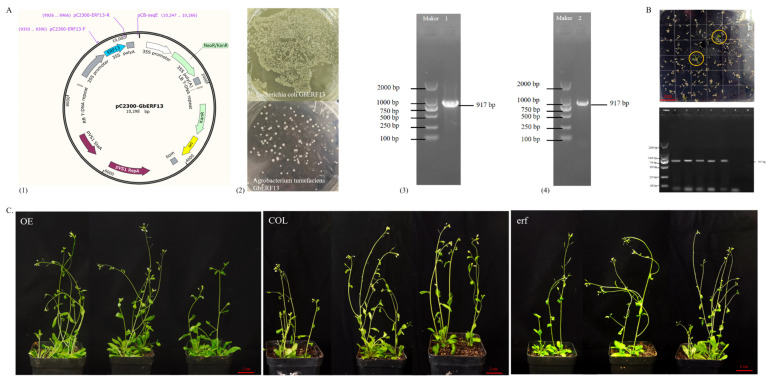
Transformation of the Sea Island cotton *GbERF13* gene and positive plants. (**A**). Cloning and vector construction of the Sea Island cotton *GbERF13* gene. Panel (1) shows the map of the pC2300-*GbERF13* CDS vector; panel (2) shows the growth of *E. coli* and Agrobacterium competent cells transformed with *GbERF13* on resistance plates; panel (3) shows the colony PCR verification bands of *E. coli*, with well 1 representing the colony spot; panel (4) shows the colony PCR verification bands of Agrobacterium, with well 2 representing the colony spot. (**B**). Selection of transformed Arabidopsis on resistance medium and PCR verification. Circle out part of the positive plant of Arabidopsis thaliana. (**C**). Overall phenotype of Arabidopsis.

To investigate the biological function of *GbERF13*, the pC2300-*GbERF13* overexpression vector was first constructed. Positive strains were obtained after transformation into *E. coli* and Agrobacterium, followed by PCR and sequencing verification (Figure 4A). Considering the developmental homology between cotton fibers and Arabidopsis trichomes, an Arabidopsis heterologous expression system was used for functional validation. After *GbERF13* was transformed into Arabidopsis, T_0_ seeds were obtained. Through resistance screening of T_1_ plants and PCR identification of T_2_ plants (Figure 4B), T_3_ homozygous positive plants were ultimately obtained for subsequent phenotypic analysis (Figure 4C).

#### 2.3.2. Phenotypic Identification of Arabidopsis Transformed with *GbERF13*

**Figure 5:**
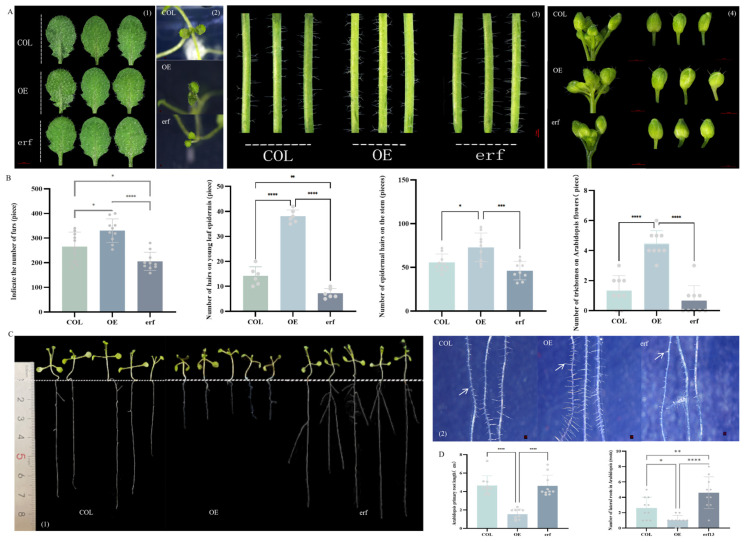
Phenotypic characterization of Arabidopsis carrying the Sea Island cotton *GbERF13* gene. (**A**). Phenotypes of mature leaves, young leaves, stems, inflorescences, and flower buds of Arabidopsis. (**B**). Statistical analysis of trichome number in different Arabidopsis organs. From left to right: differences in leaf trichome number, stem trichome number, young leaf trichome number, and floral trichome number. (**C**). Arabidopsis root development (1) and root hair growth (2). The arrow points out the root hairs. (**D**). Differences in primary root length and root hair number in Arabidopsis. Statistical analysis: One-way analysis of variance (ANOVA) and Duncan multiple range test were used for significant comparison (*p* < 0.05). Each group contains three independent biological replicates. Abbreviation definition: COL = wild-type Colombian Arabidopsis thaliana; OE = GbERF13 overexpression lines; erf = loss-of-function Arabidopsis; DPA = days after flowering. *, **, *** and **** indicate significant differences at the levels of *p* < 0.05, *p* < 0.01, *p* < 0.001 and *p* < 0.0001, respectively.

Phenotypes of T_3_ transgenic Arabidopsis plants were observed, and the numbers of trichomes on leaves, stems, and floral organs were counted and analyzed (Figure 5A). In terms of trichome development, the number of leaf trichomes in GbERF13-overexpressing transgenic Arabidopsis increased significantly by 24.5% compared with the wild type (*p* < 0.05), whereas that in the loss-of-function mutant decreased significantly by 22.6% compared with the wild type (*p* < 0.05). On young leaves, the trichome number in transgenic plants differed significantly from that in the wild type and mutant (*p* < 0.05), showing a trend consistent with that observed in mature leaves. The number of stem trichomes in transgenic Arabidopsis increased significantly by 30.9% compared with the wild type (*p* < 0.05), whereas that in the loss-of-function mutant decreased significantly by 16.3% compared with the wild type (*p* < 0.05). In floral organs, the average number of trichomes in wild-type Arabidopsis flowers was 1, whereas that in transgenic Arabidopsis increased significantly to 4, and that in the mutant was 0 (*p* < 0.05) (Figure 5B).

In terms of root development, the primary root length of GbERF13-overexpressing Arabidopsis was strongly inhibited by abscisic acid (Figure 5C), being 66.3% shorter than that of the wild type. The primary root length of the loss-of-function mutant showed no obvious change compared with the wild type, presumably because of excessive activation of *GbERF13*-mediated abscisic acid signaling. For root hair number, transgenic Arabidopsis showed a significant increase of 62.2% compared with the wild type (*p* < 0.05), whereas the loss-of-function mutant showed a significant decrease of 27.0% (*p* < 0.05) (Figure 5D), indicating that *GbERF13* positively regulates root hair development. In summary, *GbERF13* positively regulates trichome and root hair development in Arabidopsis and participates in abscisic acid-mediated inhibition of primary root elongation. At present, we have not carried out quantitative detection of cellulose and lignin, tissue staining observation, and expression analysis of cell wall synthesis-related genes such as CESA in cotton fibers and Arabidopsis tissues.

### 2.4. Effects of Exogenous ABA on GbERF13 Expression and Fiber Development

**Figure 6:**
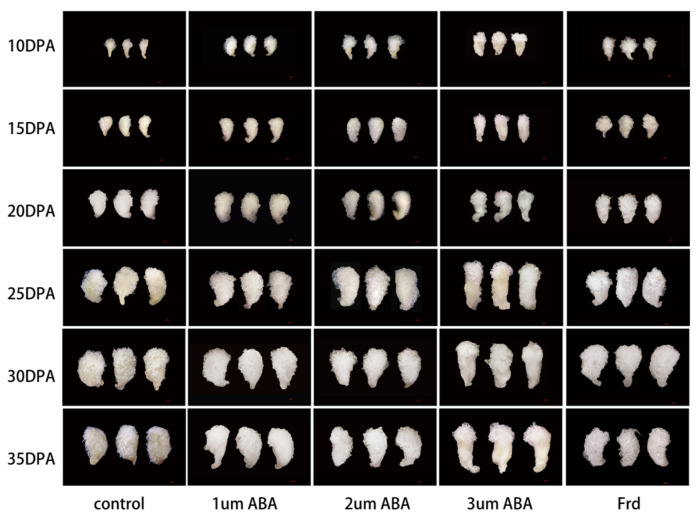
Phenotypes of island cotton ovules at different stages. In vitro-cultured Sea Island cotton ovules collected at 2 DPA were cultured continuously for 35 days, and fiber phenotypes were recorded at 10, 15, 20, 25, 30, and 35 DPA developmental stages under four ABA concentration treatments (0, 1, 2, 3 μmol/L) plus fluridone control.

**Figure 7:**
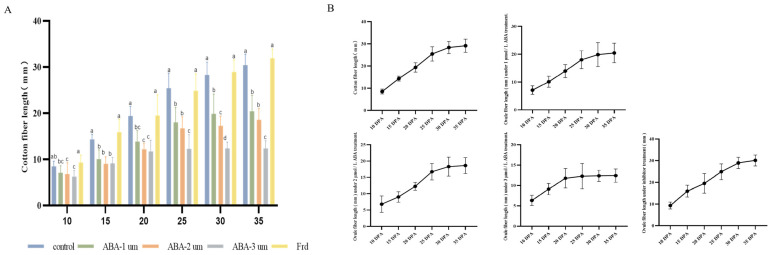
Elongation of ovule fibers after ABA application. (**A**). The fiber elongation of ovule fiber under different concentrations of ABA treatment. Different lowercase letters a, b, c, d indicate significant differences at *p* < 0.05 via Duncan’s multiple range test; shared letters mean no significant difference. (**B**). The elongation of ovule fibers in different periods under 1, 2, 3 μmol/L ABA treatment. Statistical analysis: One-way analysis of variance (ANOVA) and Duncan multiple range test were used for significant comparison (*p* < 0.05). Each group contains three independent biological replicates. Abbreviation definition: DPA = days after flowering; ABA = abscisic acid; frd = fluridone (ABA biosynthesis inhibitor).

**Figure 8:**
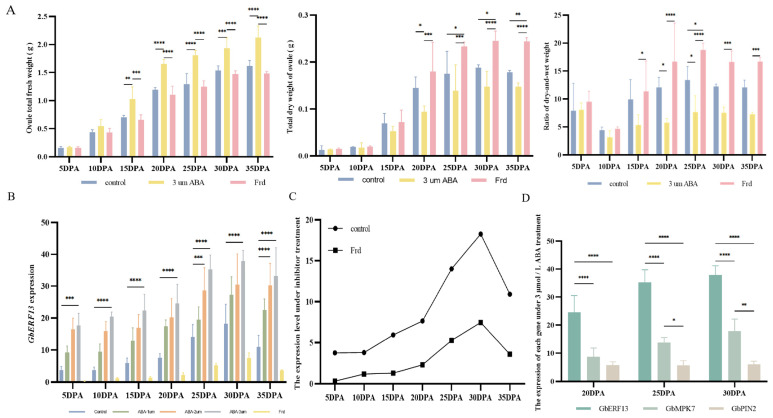
Differences in dry and fresh weight ratio and expression level of ovules. (**A**). Total fresh weight, total dry weight and ratio of dry weight to fresh weight of ovules under different treatments. (**B**). The expression of *GbERF13* on different days under different concentrations of ABA treatment was different. (**C**). The expression of *GbERF13* after adding inhibitors. (**D**). Effects of 3 μmol/L ABA treatment on the expression of *GbERF13*, *GbMPK7* and *GbPIN2* on different days. Statistical analysis: One-way analysis of variance (ANOVA) and Duncan multiple range test were used for significant comparison (*p* < 0.05). Each group contains three independent biological replicates. Abbreviation definition: DPA = days after flowering; ABA = abscisic acid; frd = fluridone (ABA biosynthesis inhibitor). *, **, *** and **** indicate significant differences at the levels of *p* < 0.05, *p* < 0.01, *p* < 0.001 and *p* < 0.0001, respectively.

Previous studies have shown that abscisic acid (ABA) mainly acts on the secondary wall thickening stage of cotton fibers and can promote the accumulation of dry matter and cellulose in the secondary wall [26]. The expression of *GbERF13* was the highest in the fiber development period (15–30 DPA), which was consistent with the secondary wall thickening stage. Therefore, the expression period of *GbERF13* is highly overlapped with the stage of ABA effect. In this study, the response of *GbERF13* to ABA signal transduction and its regulation in fiber development were investigated by applying exogenous abscisic acid (ABA) to the ovules of *Gossypium barbadense* cultured in vitro. According to the published cotton hormone treatment scheme, 3 μmol/L ABA was selected in this study, which has been proved to have significant biological effects on cotton fiber development [19,26]. The results showed that exogenous ABA treatment could adversely affect the growth and development of *G. barbadense* ovules and induce the expression of *GbERF13* in *G. barbadense* ovules (Figure 6). High concentration of ABA significantly inhibited fiber elongation and ovule dry matter accumulation. There were significant differences in fiber length between different treatment groups (*p* < 0.05) (Figure 7A). Dynamic analysis of fiber elongation showed that (Figure 7B) the fiber length of the control group continued to increase from 10 DPA to 35 DPA and reached 30.41 ± 2.25 mm at 35 DPA; however, 3 μmol/L ABA treatment significantly inhibited fiber elongation, and the elongation process almost stopped at 20 DPA. During the 25–35 DPA period, the fiber length increased by only about 0.6 mm; at 35 DPA, it decreased to 12.42 ± 1.55 mm, which was about 59.2% lower than that of the control group (*p* < 0.05) (Table 2). This inhibitory effect can be alleviated by fluridone. In addition, 3 μmol/L ABA treatment changed the material distribution pattern of ovules: taking 25 DPA as an example, the total dry weight of ovules in the treatment group decreased by about 22.6% compared with the control group, and the dry weight/fresh weight ratio decreased by about 20.1%; inhibitor treatment can reverse these changes (Figure 8A).

In order to study the effect of ABA on the expression of *GbERF13*, we detected the gene expression level under different ABA concentrations and inhibitor treatment conditions. At the early development stage of 5-day ovules, 3 μmol/L ABA treatment could increase the transcription level of *GbERF13*, indicating that ovules were highly sensitive to ABA signal transduction; 1–3 μmol/L ABA treatment could up-regulate the expression of this gene, and the induction effect increased with the increase in concentration (Figure 8B). The ABA biosynthesis inhibitor fluridone attenuated this induction effect (Figure 8C), confirming that the expression of *GbERF13* was specifically regulated by the ABA signaling pathway. Previous studies have shown that ABA can promote secondary wall thickening by regulating endogenous hormones such as auxin, and the key gene *GbPIN2* involved in polar auxin transport may antagonize ABA-induced *GbERF13* expression [26]. *GbMPK7* is positively responsive to ABA signaling, and ERF transcription factors are often used as downstream targets of *GbMPK7* signaling cascades [27]. Therefore, we compared the expression levels of these three genes at different stages of ovule fiber development: at the fiber secondary wall thickening stage of 20–30 DPA, *GbERF13* and *GbMPK7* were continuously up-regulated and showed a synchronous expression trend under 3 μmol/L ABA treatment, while the polar auxin transport gene *GbPIN2* did not show significant changes (Figure 8D). In summary, high concentrations of ABA will negatively affect the expression of *GbERF13* and *GbMPK7*.

### 2.5. Haplotype Analysis and Marker Development of the Sea Island Cotton GbERF13 Gene

**Figure 9:**
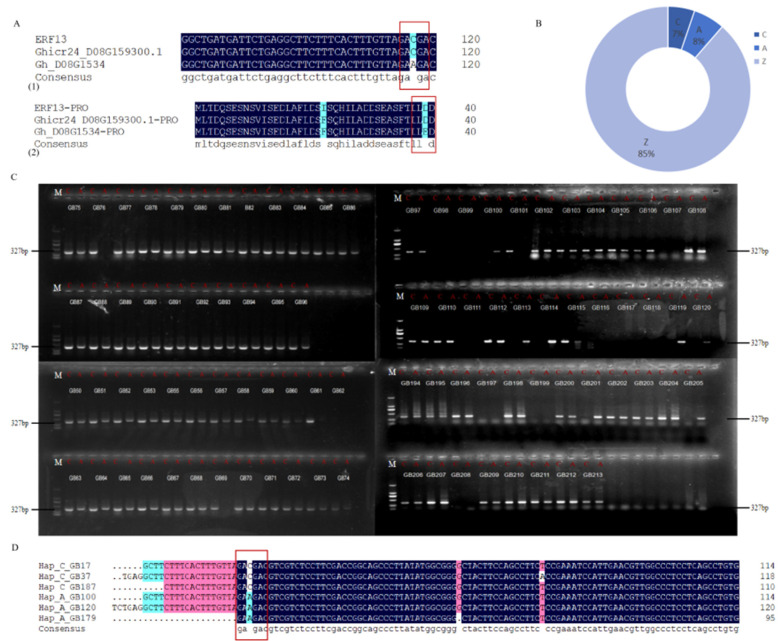
Marker development and trait association analysis of the Sea Island cotton *GbERF13* gene. (**A**). Sequence analysis of the Sea Island cotton *GbERF13* gene. Panel (1) shows nucleotide sequence analysis of the Sea Island cotton *GbERF13* gene, and panel (2) shows amino acid sequence analysis of the Sea Island cotton *GbERF13* gene. (**B**). Proportions of homozygous and heterozygous materials in the Sea Island cotton natural population. (**C**). Gel electrophoresis image of *GbERF13*-117SNP in the Sea Island cotton natural population. (**D**). Identification of *GbERF13*-117SNP in the Sea Island cotton natural population.

**Figure 10:**
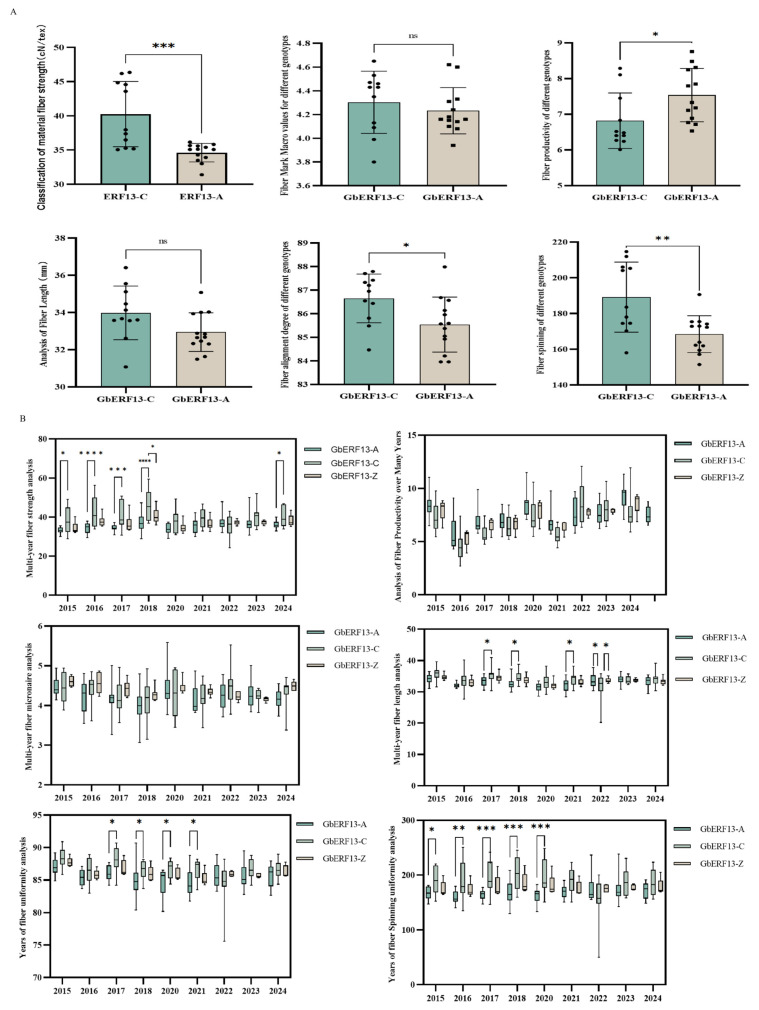
Association of *GbERF13*-SNP117 with traits. (**A**). Association between different genotypes and fiber quality. From left to right: differences in fiber strength among genotypes, differences in fiber micronaire value among genotypes, differences in fiber productivity among genotypes, differences in fiber length among genotypes, differences in fiber uniformity among genotypes, and differences in fiber spinning performance among genotypes. (**B**). Differences in fiber quality of resource materials with different genotypes in different years. From left to right: multi-year analysis of fiber strength, multi-year analysis of fiber productivity, multi-year analysis of micronaire value, multi-year analysis of fiber length, multi-year analysis of fiber uniformity, and multi-year analysis of fiber spinning performance. Statistical analysis: One-way analysis of variance (ANOVA) and Duncan multiple range test were used for significant comparison (*p* < 0.05). Each group contains three independent biological replicates. *, **, *** and **** indicate significant differences at the levels of *p* < 0.05, *p* < 0.01, *p* < 0.001 and *p* < 0.0001, respectively.

In this study, a stable A/C non-synonymous SNP locus (*GbERF13*-117SNP) was identified at 117 bp in the coding region (CDS) of *GbERF13* gene using 213 *Gossypium barbadense* germplasm materials (Figure 9A). Based on this locus, SNP markers were successfully constructed by allele-specific PCR, which could effectively distinguish homozygous genotype A and C and heterozygous genotype (Z). The population contained 213 island cotton germplasm materials, including 11 C-type homozygotes, 13 A-type homozygotes, and 174 heterozygotes (85%) (Figure 9B,C). It is speculated that the low proportion of homozygotes is related to long-term artificial breeding. The results showed that this locus could be used to identify homozygous genotypes in island cotton natural resources (Figure 9D). The multi-year average showed that the average value of type C was 6.2% higher than that of type A (Table 3). There were significant differences in fiber yield, uniformity and spinning uniformity between the two genotypes (*p* < 0.05), but there was no significant difference in fineness value (Figure 10A). The correlation analysis from 2015 to 2024 (Table A1) showed that *GbERF13*-117SNP was significantly correlated with fiber strength, length, uniformity and spinning uniformity (Figure 10B). In terms of fiber strength (except for 2022), the fiber strength of C-type materials was significantly higher than that of A-type materials in the remaining 8 years (*p* < 0.05), and the difference ranged from 2.60 to 8.71 cN/tex; in terms of fiber length, there were 6 years of data from 2015 to 2021 indicating that the fiber length of C-type materials was longer, with a difference range of 0.92 to 2.15 mm, and the differences in 2017, 2018 and 2021 were statistically significant (*p* < 0.05). From 2017 to 2021, the uniformity index of C-type material is 3.5% higher than that of A-type material on average; from 2015 to 2020, its spinning uniformity index was 4.2% higher on average. In summary, the C allele of the *GbERF13*-117SNP gene has a beneficial effect on the fiber strength, length, uniformity and spinning performance of sea island cotton, and has a pleiotropic regulation effect. The developed functional molecular markers can stably distinguish the three genotypes, with simple operation and strong specificity, and are suitable for large-scale molecular marker-assisted breeding screening.

## 3. Discussion

The AP2/ERF transcription factor superfamily is widely distributed in plants. Its core feature is that it contains an AP2 conserved domain composed of about 60–70 amino acids, which is involved in the regulation of key biological processes such as plant growth and development [8], hormone signal transduction [28] and stress response [29]. Lu et al. systematically identified the members of the *ERF* subfamily B3 group in Gossypium hirsutum and found that fragment replication and genome-wide replication events are the main driving forces for the expansion of the *ERF* family, and purification selection plays a leading role in its evolution [30]. This study found that fragment replication is also the main driving force for the expansion of the *ERF* family in *Gossypium barbadense*, indicating that the expansion mechanism of the *ERF* family is conserved among different species of Gossypium, consistent with the above research.

In the regulation of fiber development, Li et al. reported that the cotton AP2/ERF transcription factor *GhERF41* responds to ethylene signals and positively regulates the development of secondary wall of cotton fibers by regulating the expression of genes related to cell wall synthesis [31]. Unlike *GhERF41*, this study found that *GbERF13* mainly responds to ABA signal rather than ethylene signal, indicating that *GbERF* family members have undergone functional differentiation during evolution. Zhou et al.’s latest single-cell transcriptomics study revealed that in the immune response triggered by *Atflg22* in Arabidopsis, *AtERF13* acts as a central regulator to integrate immune signals, hypoxia signals, and reactive oxygen species signals to coordinate plant growth and immune balance [32]. Most *ERF* members function as ethylene-responsive factors. The classic *GhEIN3*-*GhERF*-*COBL4* module has been proven to govern cotton fiber development via ethylene signaling [14]. This study confirmed the core position of *AtERF13* in the complex signal network at the single cell level, which is logically consistent with the discovery that *GbERF13* responds to ABA signals and participates in the regulation of fiber development, suggesting that *GbERF13* may function as a signal integration node in different plant species and different biological processes. This work has not systematically compared the regulatory effects of ethylene and ABA on *GbERF13*. Nevertheless, our previous QTL mapping results indicated that the ABA pathway plays a dominant role in the regulation of fiber development related to this gene. In follow-up experiments, we will design parallel treatments to compare the responses of *GbERF13* to ethylene and ABA and further clarify the functional differentiation of this gene in different hormone signaling pathways.

In terms of functional verification, cotton *GhERF38* gene is involved in plant salt stress, drought stress and abscisic acid ABA signal response process [33]. Zhang et al. reported that the *ABI3-ERF1* module mediated ABA–auxin signal interaction to regulate lateral root formation, which provided important mechanism evidence for AP2/ERF transcription factors to integrate hormone signals and regulate plant development [34]. Through the systematic functional analysis of Arabidopsis *ERF102-ERF105* genes, Illgen et al. found that the subfamily members not only positively regulate plant cold tolerance through CBF-dependent pathways but also participate in the regulation of trichome development overexpression of *ERF102-ERF105*, which can significantly change the density and morphology of leaf trichomes [35]. In this study, it was found that the number of epidermal hairs and root hairs of Arabidopsis thaliana overexpressing *GbERF13* was significantly increased, and the main root length was significantly shortened. Together, these results suggest that *GbERF13* and its homologous genes play a key regulatory role in the ABA signaling pathway, and this function is conserved between Arabidopsis and cotton [36]. Different from *GhERF41* and *GhERF38* [31,33], *GbERF13* regulates fiber development via ABA signaling. Heterologous expression in Arabidopsis confirmed its positive effect on trichome and root hair growth. Combined with phenotypic changes and related gene expression data, we infer that *GbERF13* participates in the regulation of fiber cell wall synthesis (cellulose and lignin deposition). It should be noted that we did not quantitatively detect cellulose and lignin contents, nor perform cytochemical staining and CESA gene expression analysis in cotton fibers and Arabidopsis tissues in the current study. Combining the research progress and overall experimental arrangement, these validations will be included in our subsequent research plans to fully confirm the regulatory role of *GbERF13* in cell wall biosynthesis. This study only used the Arabidopsis thaliana heterologous system for functional verification, due to the extremely low transformation efficiency and long growth cycle of Sea Island cotton. In follow-up work, we will construct overexpression and CRISPR/Cas9 knockout vectors to perform homologous transformation in Sea Island cotton and further verify the in vivo function of *GbERF13*.

In this study, the effect of high-concentration ABA inhibiting fiber elongation can be relieved by fluridone. The expression characteristics of *GbERF13* and *GbMPK7* up-regulated synchronously under high concentration ABA treatment are consistent with the conclusion reported by Wang et al. that *GhMPK7* positively regulates ABA signaling pathway, suggesting that the two may play a synergistic role in the same signaling pathway [27]. The functional characteristics of the *ERF* gene family in plants are species conserved. The regulatory mechanism of *GbERF13* analyzed in this study is not only applicable to island cotton but also provides a theoretical reference for the quality improvement of other crops such as wheat and corn.

Based on the phenotypic characteristics of transgenic Arabidopsis and the regulatory characteristics of the ABA pathway, we inferred that *GbERF13* participates in the cell wall development of cotton fibers and epidermal cells. However, this conclusion is only supported by phenotypic evidence at this stage. We have not supplemented the detection of main cell wall components (cellulose, lignin), cytochemical staining, and expression verification of key cell wall synthesis genes including CESA in both Sea Island cotton and Arabidopsis. These experimental deficiencies are mainly limited by the current research progress. In follow-up studies, we plan to (1) determine the content of cellulose and lignin in cotton fibers and Arabidopsis vegetative tissues; (2) perform histochemical staining to observe the distribution of cell wall components; (3) detect the expression levels of CESA and other cell wall-related genes via qRT-PCR, so as to further confirm the regulatory effect of *GbERF13* on cell wall biosynthesis.

In terms of molecular marker development, some scholars have carried out SNP marker mining of cotton fiber quality and confirmed that functional SNP loci can effectively distinguish germplasm genotypes with excellent fiber quality [37]; there are also studies on the development of tightly linked InDel molecular markers for the QTL of fiber strength in island cotton, and it is clear that the markers have a stable genetic effect on fiber quality traits [38]; in addition, QTL meta-analysis of cotton fiber quality-related traits was conducted to obtain key candidate genes regulating fiber development [39]. Guo et al. identified an overlapping interval closely related to fiber length on the D11 chromosome of upland cotton. Haplotype analysis showed that the fiber length of the germplasm carrying the Hap1 haplotype was significantly longer than that of the Hap2 haplotype [40]. In this study, the markers developed based on the A/C non-synonymous SNP locus at 117 bp in the CDS region of *GbERF13* gene had trait association specificity. The developed allele-specific PCR marker can be directly applied to cotton molecular marker-assisted selection (MAS). In practical breeding work, researchers can use this marker to conduct rapid genotyping of breeding populations and germplasm resources; distinguish AA, AC, and CC three genotypes at the seedling stage; and directly screen individual plants carrying the favorable C allele with excellent fiber quality. This marker enables early-stage selection, saves field identification time, and supports large-scale cotton germplasm screening and hybrid parent selection. Further exploration of the protein interaction network and molecular mechanism of *GbERF13* will be conducted in follow-up studies.

In this study, 213 natural germplasms of *Gossypium barbadense* were genotyped. It was found that the proportion of heterozygous individuals was as high as 85%, and the two homozygous genotypes of A and C accounted for only 11.3%. This phenomenon is a typical feature of the natural germplasm population of Sea Island cotton. In the process of long-term domestication and modern breeding of Sea Island cotton, breeders always aim at comprehensive fiber quality and agronomic traits. The C excellent allele linked to high-quality fiber traits has been continuously applied to cross-breeding, and the hybrid offspring naturally maintain a heterozygous state, resulting in a continuous accumulation of heterozygous genotypes in the retained germplasm. In addition, *G. barbadense* is an allotetraploid crop with complex genetic background and certain outcrossing characteristics, which further improves the heterozygosity of germplasm, resulting in the scarcity of homozygous individuals in conventionally preserved populations [36].

In general, the uneven distribution of genotypes in this population is the result of long-term artificial selection, hybrid breeding mode and the genetic characteristics of sea island cotton. Although the number of homozygous materials is limited, the association between *GbERF13*-117SNP locus and fiber quality traits is still reliable in combination with years of phenotypic identification results.

In summary, this study clarified the evolutionary characteristics of the *ERF* family in *Gossypium barbadense*, revealed the biological function of *GbERF13* in regulating fiber development by responding to ABA signals, and developed functional markers that can be used for molecular breeding. Whether there is a direct protein interaction or phosphorylation regulation relationship between *GbERF13* and *GbMPK7*, and the specific mechanism of non-synonymous SNP affecting fiber quality, still needs to be further clarified by subsequent experiments. Additionally, since the subcellular localization of *GbERF13* is currently limited to bioinformatic prediction (Table A1), we will carry out GFP fluorescence localization experiments in our next research to confirm its actual localization.

## 4. Materials and Methods

### 4.1. Experimental Materials

The Sea Island cotton materials used in this study were Pima S-7, 5917, and Xinnongda Cotton H1, and Arabidopsis thaliana (Columbia ecotype) was also used. All materials were provided by the Xinjiang Key Laboratory of Crop Biological Breeding, Xinjiang Agricultural University. Cotton field samples from 15 plants with consistent growth were selected for each biological replicate, and 10 healthy seedlings were used for each biological replicate in the Arabidopsis thaliana test. Pima S-7 and 5917 were used for gene expression analysis and gene cloning. Xinnongda Cotton H1 was selected for the ovule in vitro culture system; it is a Sea Island cotton germplasm preserved in the laboratory and shows a high ovule survival rate and stable fiber induction in vitro. A recombinant inbred line population constructed previously from this combination had successfully mapped multiple QTLs associated with fiber quality, providing reliable genetic material support for the screening and functional analysis of the candidate gene *GbERF13* in this study. The plant expression vector was pC2300S. Escherichia coli DH5α and Agrobacterium tumefaciens GV3101 competent cells were used. Restriction endonucleases, seamless cloning kits, RNA extraction kits, DNA recovery and purification kits, reverse transcription kits, qPCR kits, and other reagents were commercially available. A natural population of 213 Sea Island cotton accessions was used for haplotype analysis and marker development.

### 4.2. Total RNA Extraction and cDNA Synthesis from Sea Island Cotton

A total RNA extraction kit for polysaccharide- and polyphenol-rich plant tissues was used to extract total RNA from key tissues and organs of Sea Island cotton at different growth and developmental stages, including roots, stems, leaves, flowers, and fibers (5 DPA, 10 DPA, 20 DPA, 25 DPA, 30 DPA, and 35DPA). Three biological replicates were established for each tissue or organ. Total RNA was also extracted from ovules at different stages under different treatments. First-strand cDNA was synthesized according to the instructions of the reverse transcription kit and used for tissue-specific expression analysis. The primers used for expression analysis are listed in Table 4.

### 4.3. Identification and Expression Analysis of the GbERF Family in Sea Island Cotton

Based on the hidden Markov model of the AP2/ERF domain in the Pfam database (Pfam accession: cd00018), HMMER 3.0 was used to search the whole-genome protein sequences of Sea Island cotton, *Gossypium raimondii*, upland cotton (*Gossypium hirsutum* L.), and Asiatic cotton (*Gossypium arboreum* L.) to obtain candidate ERF protein sequences. Conserved domains were verified using the online SMART and Pfam tools, and redundant or incomplete sequences were removed to identify *ERF* family members in each cotton species. Multiple sequence alignment of ERF protein sequences was performed using MEGA 12.0, and a phylogenetic tree was constructed using the Neighbor-Joining method with 1000 bootstrap replicates. The tree was visualized and refined using the online iTOL tool. Conserved motif analysis was performed using the MEME online analysis tool, with the maximum number of motifs set to 10 and motif length set to 6–50 amino acids. Chromosomal localization and intra- and interspecific collinearity analyses were conducted using TBtools-II (v2.303) to clarify gene duplication and evolutionary characteristics. Based on qRT-PCR data from different tissues of Sea Island cotton (root, stem, leaf, and flower) and different fiber developmental stages (5 DPA, 10 DPA, 20 DPA, 25 DPA, 30 DPA, and 35DPA), the expression patterns of *GbERF* family members were analyzed. Three biological replicates were included for each sample, and GraphPad Prism 9.0 and IBM SPSS Statistics 31.0 (IBM Corp., Armonk, NY, USA) were used for statistical analysis and visualization.

### 4.4. Cloning and Transformation of the Sea Island Cotton GbERF13 Gene

The CDS sequence was obtained according to the gene accession number Gbar_D08G016020.1, and specific primers were designed using SnapGene (Table 5). PCR amplification was performed using cDNA as the template. The products were recovered from gels and purified, then ligated into the pC2300S vector through double digestion with *Kpn* I and *Bam*H I followed by seamless cloning to construct the pC2300-*GbERF13* overexpression vector. The recombinant vector was transformed into *E. coli* DH5α by heat shock. After verification by colony PCR and sequencing, the vector was introduced into A. tumefaciens GV3101 using the freeze–thaw method [41]; the confirmed positive strains were retained for subsequent transformation to carry out heterologous overexpression of *GbERF13*. Because the stable genetic transformation technology of island cotton is difficult and the transformation efficiency is extremely low, this study selected Arabidopsis thaliana as a heterologous model plant to carry out preliminary functional identification of the cotton gene. Arabidopsis was transformed using the floral dip method [9]. T_0_ seeds were harvested, and positive seedlings were selected on MS resistance medium containing kanamycin and then transplanted into nutrient soil. DNA was extracted from T_2_ plants for PCR verification, and T_3_ homozygous transgenic lines were obtained by further cultivation for phenotypic analysis. For Arabidopsis phenotypic analysis, 10 individual plants were selected per biological replicate, with three biological replicates in total.

### 4.5. In Vitro Culture and Phenotypic Analysis of Sea Island Cotton Ovules

On the day of flowering at full bloom, cotton bolls were marked as 0 DPA, and 2 DPA bolls were collected. Ovules were excised under sterile conditions according to the in vitro ovule culture method described by Shi Huiyun et al. [42]. Ovules were placed on media supplemented with different concentrations of ABA (0, 1, 2, and 3 μmol/L) and on medium supplemented with fluridone under the 3 μmol/L ABA treatment, followed by dark incubation at 31 °C for 35 DPA. The treatment without ABA was used as the control, with three replicates for each treatment. Each culture dish contained 20 uniform ovules per biological replicate; three independent biological replicates were set for all morphological and physiological index measurements.

Ovule and fiber development was observed every 5 DPA, and fiber length was measured from 15 to 35 DPA. Ovule fresh weight was recorded, and dry weight was determined after drying at 80 °C to constant weight. Gene expression levels were detected by qRT-PCR, with *UBQ7* used as the internal reference gene. Relative expression levels were calculated using the 2^−ΔΔCt^ method, with three biological replicates and three technical replicates for each sample.

### 4.6. Haplotype Analysis and Marker Development for GbERF13

DNAMAN was used for sequence alignment to screen SNP variations in the coding region and regulatory region of *GbERF13* gene. Based on these SNP loci, functional molecular markers were developed by allele-specific PCR technology (Table 6). Genotyping experiments were carried out with three technical replicates for each sample. The allele-specific PCR primers introduced mismatched bases at the 3′ end to improve amplification specificity; in this study, the PCR system and reaction procedure were optimized repeatedly to ensure the stability of typing. The PCR system contained 2×Taq-HS mixture, specific primers and DNA template. The amplification procedure was 35 cycles after pre-denaturation at 94 °C; the amplified products were detected by agarose gel electrophoresis, and the three genotypes of AA, CC and AC could be stably distinguished. Phenotypic data of fiber quality in nine environments from 2015 to 2024 were collected, and variance analysis was performed using software IBM SPSS Statistics 31.0 (IBM Corp., Armonk, NY, USA) to clarify the association effects between different haplotypes and fiber strength, length and uniformity.

## 5. Conclusions

In this study, 161 nonredundant *GbERF* family members were systematically identified across the Sea Island cotton genome. These genes were unevenly distributed on chromosomes and showed high homology with the distribution patterns of diploid ancestral cotton species. Phylogenetic analysis divided *GbERF* members into nine groups, among which *GbERF13* belonged to the Group V branch. All *GbERF* family proteins contained a typical conserved AP2 domain. Motif 1 and Motif 2 were shared by all members and constituted the core region of the AP2 domain, whereas Motifs 3, 5, and 6 showed branch-specific distributions and formed the molecular basis for functional differentiation of the *ERF* family. Collinearity analysis showed that segmental duplication was the major driver of *ERF* family expansion in Sea Island cotton, with only limited gene loss and chromosomal rearrangement during polyploidization. Expression pattern analysis showed that the family members had significant tissue specificity, and some genes showed specific high expression during fiber development at 10–25 DPA. The Sea Island cotton *GbERF13* gene had a full-length CDS of 617 bp and encoded 188 amino acids. It encoded an unstable hydrophilic protein localized to the nucleus, and its secondary structure was dominated by random coils, accounting for 77.37%. This gene was specifically and highly expressed in fibers, and its expression continued to increase significantly at 15–30 DPA, closely coinciding with the key transition from rapid fiber elongation to secondary wall thickening. Hormone response experiments confirmed that *GbERF13* is a key ABA-responsive gene. Its expression was induced by exogenous ABA, and the ABA inhibitor fluridone suppressed this induction effect. In vitro ovule culture showed that 3 μmol/L high-concentration ABA significantly inhibited fiber elongation, causing fiber elongation to be almost arrested after 20 DPA, while also reducing ovule dry matter accumulation and increasing ovule water content. This inhibitory effect could be alleviated by fluridone. Arabidopsis plants overexpressing *GbERF13* had 24.5% more trichomes than the wild type, 62.2% more root hairs, and 66.3% shorter primary roots. *GbERF13* and *GbMPK7* jointly participated in the regulation of cotton fiber development and showed synchronized up-regulation under ABA treatment. During the fiber secondary wall thickening stage (20–30 DPA), the expression levels of both genes continued to increase, whereas the polar auxin transport gene *GbPIN2* showed no significant change. An A/C nonsynonymous SNP (*GbERF13*-117SNP) was identified at 117 bp in the CDS region of *GbERF13*. Genotyping of 213 Sea Island cotton accessions showed that heterozygotes accounted for 85%, the C-type homozygous genotype for 5.2%, and the A-type homozygous genotype for 6.1%. Multi-year and multi-location association analysis showed that C-genotype materials were significantly superior to A-type materials in fiber strength, length, uniformity, and spinning evenness. Fiber strength increased by an average of 6.2%, the maximum difference in fiber length reached 2.15 mm, the uniformity index increased by an average of 3.5%, and the spinning evenness index increased by an average of 4.2%; there was no significant difference in micronaire value between the two genotypes. Based on this SNP locus, an allele-specific codominant molecular marker was successfully developed, which can stably distinguish the three genotypes and is suitable for large-scale breeding screening.

## Figures and Tables

**Table 1:** Functional annotation of conserved motifs.

Motif	Core Functional Region	Biological Role
Motif 1	Core conserved region of the AP2 domain	This motif constitutes the core framework of the characteristic AP2 DNA-binding domain of the AP2/ERF family and is responsible for specifically binding cis-acting elements, such as the GCC-box (AGCCGCC), in target gene promoter regions. It is the core element through which ERF transcription factors exert transcriptional regulatory functions.
Motif 2	N-terminal conserved region of the AP2 domain	Together with Motif 1, this motif forms the complete AP2 domain, maintains the three-dimensional conformation of the DNA-binding domain, and enhances binding affinity to cis-elements. It is a characteristic conserved motif for *ERF* family classification.
Motif 4	Transcriptional regulatory functional region	This motif is mostly a core element of transcriptional activation or repression domains. By recruiting transcriptional coactivators or corepressors, it regulates the transcriptional efficiency of downstream target genes and participates in transcriptional regulatory networks related to plant hormone signaling and stress responses.
Motif 5	Key motif for functional differentiation	Located downstream of the AP2 domain, this motif is an important marker of functional differentiation among *ERF* subfamily members. Its presence or absence in different subfamilies is directly related to tissue-specific expression and stress response specificity.
Motif 3, Motif 6, Motif 7	Transcriptional regulatory/protein interaction region	These motifs are mostly transcriptional activation domains (ADs), nuclear localization signals (NLSs), or protein interaction motifs. They mediate interactions between ERF proteins and other transcription factors or kinases and participate in the cascade amplification and regulation of signaling pathways.
Motif 8, Motif 9, Motif 10	Subfamily-specific functional motif	These motifs are specific to particular *ERF* subfamilies and determine tissue expression specificity and stress response types, such as responses to drought, high salinity, and ABA. They constitute the molecular basis of functional differentiation in the *ERF* family.

**Table 2:** Fiber length of ovules at different stages under different treatments.

Days (DPA)	Treatment Group	Mean (mm)	Mean ± SD
10	Control	8.51	8.51 ± 1.04 ab
	1 μmol/L ABA	7.11	7.11 ± 1.46 bc
	2 μmol/L ABA	6.81	6.81 ± 2.38 c
	3 μmol/L ABA	6.32	6.32 ± 1.2 c
	+Frd	9.33	9.33 ± 1.52 a
15	Control	14.35	14.35 ± 1.05 a
	1 μmol/L ABA	10.12	10.12 ± 1.87 b
	2 μmol/L ABA	9.00	9.00 ± 1.51 b
	3 μmol/L ABA	9.11	9.11 ± 1.31 b
	+Frd	15.95	15.95 ± 2.58 a
20	Control	19.37	19.37 ± 2.02 a
	1 μmol/L ABA	13.93	13.93 ± 2.22 bc
	2 μmol/L ABA	12.26	12.26 ± 1.15 c
	3 μmol/L ABA	11.78	11.78 ± 2.26 c
	+Frd	19.52	19.52 ± 4.28 a
25	Control	25.46	25.46 ± 3.06 a
	1 μmol/L ABA	18	18 ± 3.07 b
	2 μmol/L ABA	16.74	16.74 ± 2.39 b
	3 μmol/L ABA	12.28	12.28 ± 2.93 c
	+Frd	24.91	24.91 ± 3.47 a
30	Control	28.36	28.36 ± 2.59 a
	1 μmol/L ABA	19.87	19.87 ± 4.05 b
	2 μmol/L ABA	17.31	17.31 ± 1.97 c
	3 μmol/L ABA	12.37	12.37 ± 1.31 d
	+Frd	28.96	28.96 ± 2.44 a
35	Control	30.41	30.41 ± 2.25 a
	1 μmol/L ABA	20.45	20.45 ± 3.3 b
	2 μmol/L ABA	18.63	18.63 ± 2.31 b
	3 μmol/L ABA	12.42	12.42 ± 1.55 c
	+Frd	31.94	31.94 ± 1.86 a

Note: Different lowercase letters a, b, c, d indicate significant differences at *p* < 0.05 via Duncan’s multiple range test; shared letters mean no significant difference.

**Table 3:** Fiber strength performance of two genotypes across different years.

Year	Genotype	Sample Size	Mean	Standard Deviation	Skewness	Kurtosis	Maximum	Minimum	Range	Coefficient of Variation
2015	C	11	38.30	6.74	0.23	−1.44	49.10	28.90	20.20	0.18
2015	A	13	33.36	1.58	−0.49	−0.04	35.65	30.10	5.55	0.05
2016	C	11	42.64	8.31	0.51	−1.29	56.33	33.96	22.37	0.19
2016	A	13	34.50	2.62	−0.57	−0.82	37.92	29.53	8.39	0.08
2017	C	11	41.63	7.42	0.02	−1.88	50.75	30.74	20.01	0.18
2017	A	13	34.50	1.70	−0.38	0.31	37.20	31.04	6.16	0.05
2018	C	11	45.90	8.54	0.53	−1.26	59.40	36.88	22.52	0.19
2018	A	13	37.19	4.81	0.60	0.67	47.34	28.99	18.35	0.13
2020	C	11	38.42	6.22	0.41	−0.82	49.29	30.87	18.42	0.16
2020	A	13	34.01	2.60	−0.10	−0.71	37.44	29.11	8.33	0.08
2021	C	11	38.75	5.18	0.07	−1.44	46.65	31.24	15.41	0.13
2021	A	13	35.31	3.62	0.20	−0.67	42.24	29.99	12.25	0.10
2022	C	11	35.63	5.51	−0.64	0.33	43.03	24.22	18.81	0.15
2022	A	13	37.34	3.86	1.71	4.48	47.92	32.09	15.84	0.10
2023	C	11	40.24	5.44	0.85	0.93	52.07	33.52	18.54	0.14
2023	A	13	37.64	5.11	1.54	2.46	50.10	30.76	19.34	0.14
2024	C	11	40.49	5.53	0.07	−2.17	46.85	33.98	12.87	0.14
2024	A	13	36.06	1.83	0.53	0.46	39.95	32.92	7.03	0.05

**Table 4:** Primers used for qRT-PCR.

Primer Name	Primer Sequence
Gh*UBQ7*-F	GAAGGCATTCCACCTGACCAAC
Gh*UBQ7*-R	CTTGACCTTCTTCTTCTTGTGCTTG
YG-*MPK7*-F	GCTGAAGTACCTCCACTCG
YG-*MPK7*-R	TCCTACCGACCAAACATCAATG
YG-*PIN2*-F	CAGTGGGTGTGTTGGTATGT
YG-*PIN2*--R	TGGGATCATGGTTAATGGAAGG
YG-*ERF13*-F2	GGTGGGTAGTGGGAAAGGG
YG-*ERF13*-R2	GCCACGAATCTTGAAAGCGG

**Table 5:** Primers used for cloning and vector construction of the Sea Island cotton *GbERF13* gene.

Primer Name	Sequence (5′–3′)	Purpose
*pC2300-ERF13*-F	gagctttcgcgagctcggtaccATGTTGACCGATCAGTCTG	Cloning and vector construction
*pC2300-ERF13*-R	gcaggtcgactctagaggatccTTATGGCTTCGGCATAGTG	
pCB-seqE	GCACCCCAGGCTTTACACTT	Sequencing

Note: Lowercase nucleotide sequences represent protective bases and restriction enzyme sites.

**Table 6:** Allele-specific marker primers.

Primer Name	Primer Sequence
DBX-13-F-A	GCTTCTTTCACTTTGTTAGAA
DBX-13-F-C	GCTTCTTTCACTTTGTTAGAC
DBX-13-R.2	TCAAGTGAGGGAAATTGAG

## Data Availability

The datasets generated and analyzed during the current study are available from the corresponding author upon reasonable request. The sequence data of *GbERF13* have been deposited in the NCBI GenBank database under the accession number Gbar_D08G016020.1. All other data supporting the findings of this study are included in the article.

## Abbreviations

The following abbreviations are used in this manuscript:

DPA	Days post anthesis
ABA	Abscisic acid
ERF	Ethylene Response Factor
AP2	APETALA2
SNP	Single Nucleotide Polymorphism
qRT-PCR	Quantitative real-time PCR
CDS	Coding sequence
QTL	Quantitative trait locus
MPK	Mitogen-activated protein kinase
Frd	Fluridone

## Appendix A

**Table A1:** Summary of bioinformatics analysis of the Sea Island cotton *GbERF13* gene.

Gene Name	Length	Molecular Weight	Isoelectric Point	Subcellular Localization	Alpha Helix (Hh)	Extended Strand (Ee)	Random Coil (Cc)
*GbERF13*	617 bp	20,746.27 Da	9.15	Nucleus	17.37%	5.26%	77.37%

**Table A2:** Fiber length performance of two genotypes across different years.

Year	Genotype	Sample Size	Mean	Standard Deviation	Skewness	Kurtosis	Maximum	Minimum	Range	Coefficient of Variation
2015	C	11	35.43	2.20	0.02	0.56	39.64	31.55	8.09	0.06
2015	A	13	34.21	1.50	−0.61	0.19	36.46	31.03	5.44	0.04
2016	C	11	33.65	3.18	0.26	1.65	40.21	27.66	12.55	0.09
2016	A	13	32.02	0.76	0.93	0.38	33.72	31.10	2.61	0.02
2017	C	11	35.46	2.83	0.48	1.11	40.95	30.41	10.54	0.08
2017	A	13	33.32	1.57	−0.42	−0.70	35.75	30.54	5.20	0.05
2018	C	11	34.72	1.94	0.58	0.81	38.76	31.77	6.99	0.06
2018	A	13	32.57	1.87	1.10	2.66	37.27	29.84	7.43	0.06
2020	C	11	33.23	2.55	0.47	0.23	38.19	29.14	9.05	0.08
2020	A	13	31.46	1.45	−0.54	−0.25	33.48	28.61	4.87	0.05
2021	C	11	33.84	2.19	0.30	0.43	38.24	30.40	15.53	0.13
2021	A	13	32.17	2.05	−0.32	−0.78	35.40	28.39	7.01	0.06
2022	C	11	31.48	4.15	−2.20	6.08	35.75	20.22	15.53	0.13
2022	A	13	33.61	2.32	0.70	−0.28	37.79	30.37	7.42	0.07
2023	C	11	33.75	1.24	0.38	−1.38	35.73	32.30	3.43	0.04
2023	A	13	33.97	1.41	−0.53	1.59	36.52	30.76	5.76	0.04
2024	C	11	34.14	2.35	0.69	1.30	39.14	30.33	8.81	0.07
2024	A	13	33.22	1.81	−1.01	0.17	35.41	29.45	5.96	0.05
